# Recombinant chromosome 4 in two fetuses - case report and literature review

**DOI:** 10.1186/s13039-018-0393-1

**Published:** 2018-08-22

**Authors:** Yi Wu, Yanlin Wang, Shi Wu Wen, Xinrong Zhao, Wenjing Hu, Chunmin Liu, Li Gao, Yan Zhang, Shan Wang, Xingyu Yang, Biwei He, Weiwei Cheng

**Affiliations:** 1 Prenatal Diagnostic Center, International Peace Maternity & Child Health Hospital, School of Medicine, Shanghai JiaoTong University, Shanghai, China; 2 OMNI Research Group, Department of Obstetrics and Gynecology, Faculty of Medicine, University of Ottawa, Ottawa, Canada; 3 Clinical Epidemiology Program, Ottawa Hospital Research Institute, Ottawa, Canada; 4 School of Epidemiology & Public Health, Faculty of Medicine, University of Ottawa, Ottawa, Canada; 5 Department of Reproductive Genetics, International Peace Maternity & Child Health Hospital, School of Medicine, Shanghai Jiao Tong University, Shanghai, China; 6 Central laboratory, International Peace Maternity & Child Health Hospital, Shanghai JiaoTong University School of Medicine, Shanghai, China

**Keywords:** Recombinant (4) syndrome, Congenital heart disease, Prenatal diagnosis, Rare genetic disorder

## Abstract

**Background:**

Recombinant chromosome 4 syndrome (rec 4 syndrome) is a rare genetic disorder, predominately resulting from a parental pericentric inversion of chromosome 4. To date, a total of 18 cases of rec (4) syndrome were published in literature. We report the first kindred of rec (4) syndrome analyzed using copy number variation sequencing (CNV-seq).

**Results:**

A woman with two adverse fetal outcomes was described in the present study. The first fetus presented with severe intrauterine growth restriction, hyposarca, hydrothorax and ascites. The CNV-seq revealed a dup 4q and del 4p. The second fetus presented with cardiovascular disease of ventricular septal defect, overriding aorta and persistent trunk. The CNV-seq revealed a dup 4p and del 4q. We collected 18 rec (4) cases through literature review. Genotype-phenotype correlation analysis was also performed.

**Conclusion:**

Recombinant 4 syndrome is a rare genetic disorder. It should be divided into two categories according to the alternative recombinant types. The clinical manifestations of rec (4) cases with dup 4q and del 4p are consistent with the Wolf-Hirschhorn syndrome. For cases harboring dup 4p and del 4q, the high incidence of congenital heart disease is prominent.

## Background

Imbalances of chromosome 4 include various types of constitutional abnormalities, including Wolf-Hirschhorn syndrome (WHS, 4p- syndrome) [1], 4q- syndrome [2], dup 4p syndrome [3] and dup 4q syndrome [4]. Amongst the constitutional anomalies of chromosome 4, the rarest condition is the “recombinant chromosome 4 syndrome”-rec (4) syndrome, with concomitant deletion and duplication on the same chromosome 4, resulting from a pericentric inversion in a parent [2]. The genetic condition of recombinant (4) syndrome is so rare that its incidence in population has not been estimated. This condition was initially called dup 4p syndrome, because only large duplications of 4p were observed by the conventional karyotyping, while the small 4q terminal deletions were missed due to low resolution [5]. More recently, molecular techniques have led to the discovery of several dup 4p syndrome cases which were actually inverted 4p duplication combined with the 4q terminal deletions. Hence, some authors also called it “inv dup del 4” [6].

Here, we report on a woman who had two adverse pregnancy outcomes, with two different types of chromosome 4 recombinants. The CNV-seq results of both fetuses revealed the same breakpoints on the chromosome 4, involving 4p15.2 and 4q32.3. One fetus was rec (4) dup (4p) del (4q), the other was rec (4) dup (4q) del (4p). The size of deletion and duplication distal to breakpoints are very similar to each other (both approximately 23 Mb). The breakpoint of 4q32.3 on the long arm of chromosome 4 has never been reported among rec (4) cases. Also, a genotype-phenotype correlation study was performed between the present cases and previously reported cases of rec (4), to further delineate the relationship of specific chromosomal breakpoints with clinical features.

## Methods

### Patients

#### Fetus 1

**Fig. 1:**
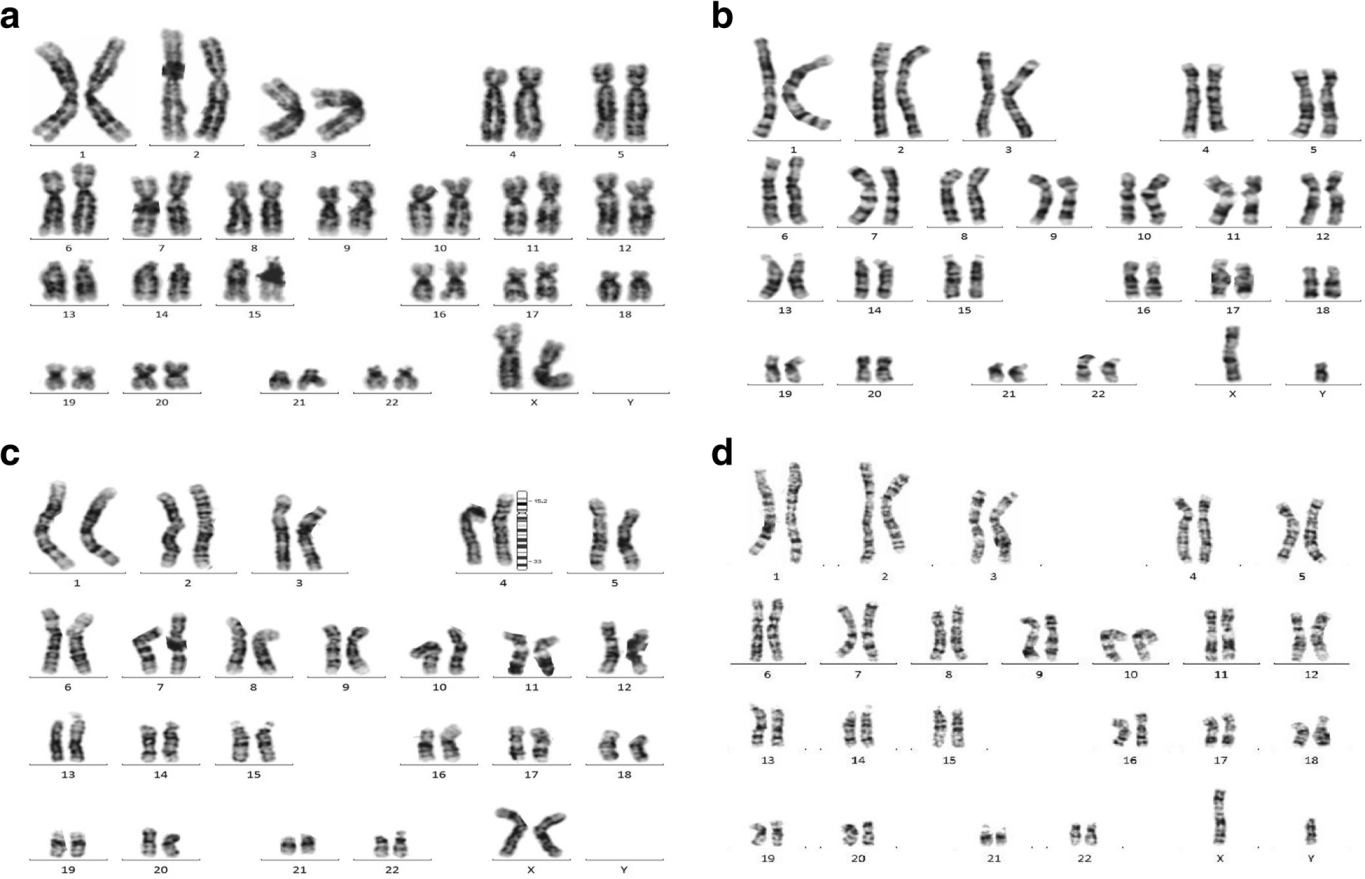
karyotypes of fetuses and parents. **a** karyotyping of the first fetus; **b**: karyotyping of the second fetus; **c** and (**d**): results of karyotyping of the parents, respectively

**Fig. 2:**
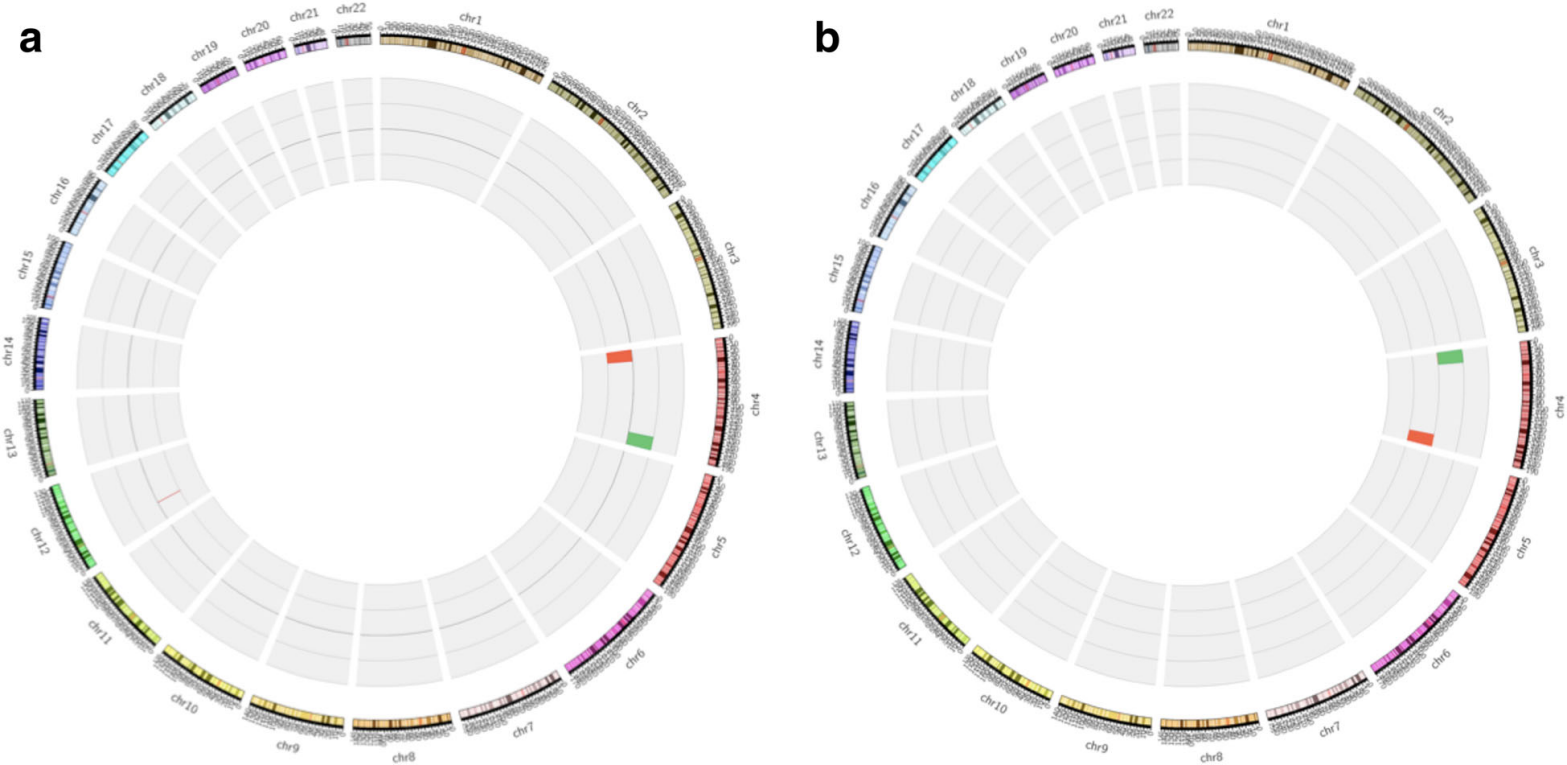
CNV seq results of two fetuses. **a** CNV-seq result of fetus 1: arr [GRCh37] 4p15.2p16.3 (4001–23,300,000) × 1, 4q32.3 q35.2 (167040001–190,940,000) × 3. **b** CNV-seq result of fetus 2: arr [GRCh37] 4p15.2 p16.3(40001–23,320,000) × 3, 4q32.3 q35.2 (167040001–190,940,000) × 1

**Fig. 3:**
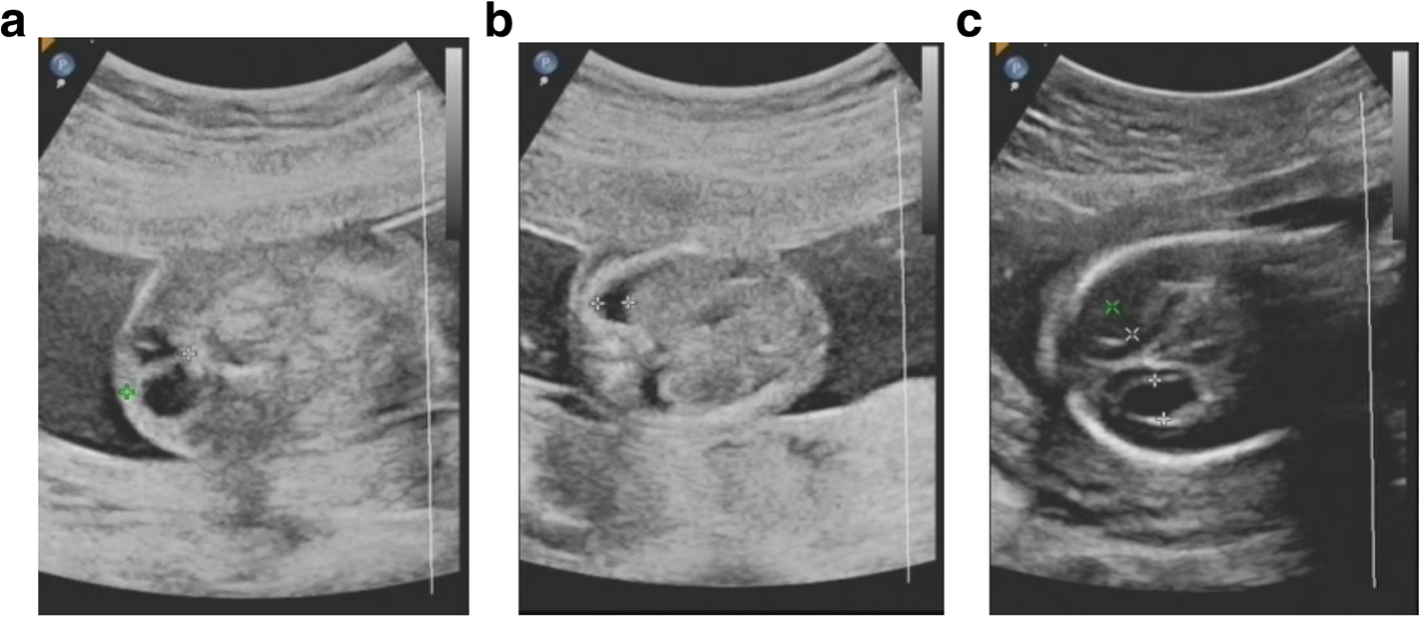
ultrasound findings of the first fetus. **a** increased nuchal fold with septation; **b** hydrothorax; **c** bilateral mild ventriculomegaly

A 33-year-old G1P0 pregnant woman was referred to the Prenatal Diagnosis center of International Peace Maternity & Child Health Hospital at 11 weeks of gestation due to increased fetal nuchal translucency (NT 10 mm). The couple was non-consanguineous and this pregnancy was naturally conceived. CVS sampling and cytogenetic analysis were suggested but declined. At 16 weeks, ultrasound findings revealed severe fetal hyposarca and intrauterine growth restriction (IUGR), with the fetal biometry <10th centile. Parents then accepted amniocentesis. After signing the informed consent, both conventional karyotyping and copy number variation sequencing (CNV-seq) were performed on amniocytes. Parental karyotypes were also tested. There were no gross abnormal findings in fetal and parental karyotypes (Fig. 1a, c and d). However, the fetal CNV-seq results were abnormal: arr [GRCh37] 4p15.2p16.3 (4001–23,300,000) × 1, 4q32.3q35.2 (167040001–190,940,000) × 3(Fig. 2a). The fetal karyotyping was actually 46, XX, der (4), (qter→q32.3::p15.2 → qter), according to the ISCN (2016). The fetal chromosomal abnormalities were overlooked due to the poor quality of G-banding and low resolution. Ultrasound findings at 21 weeks revealed IUGR, hydrothorax (4 mm) and ascites (5 mm), increased nuchal fold (NF 12.7 mm, septation was seen), hydrothorax and bilateral mild ventriculomegaly (left 7.1 mm and right 6.0 mm) (seen in Fig. 3a, b and c). Fetal echocardiography was normal. Pregnancy was terminated at 24 weeks by the parental request.

#### Fetus 2

**Fig. 4:**
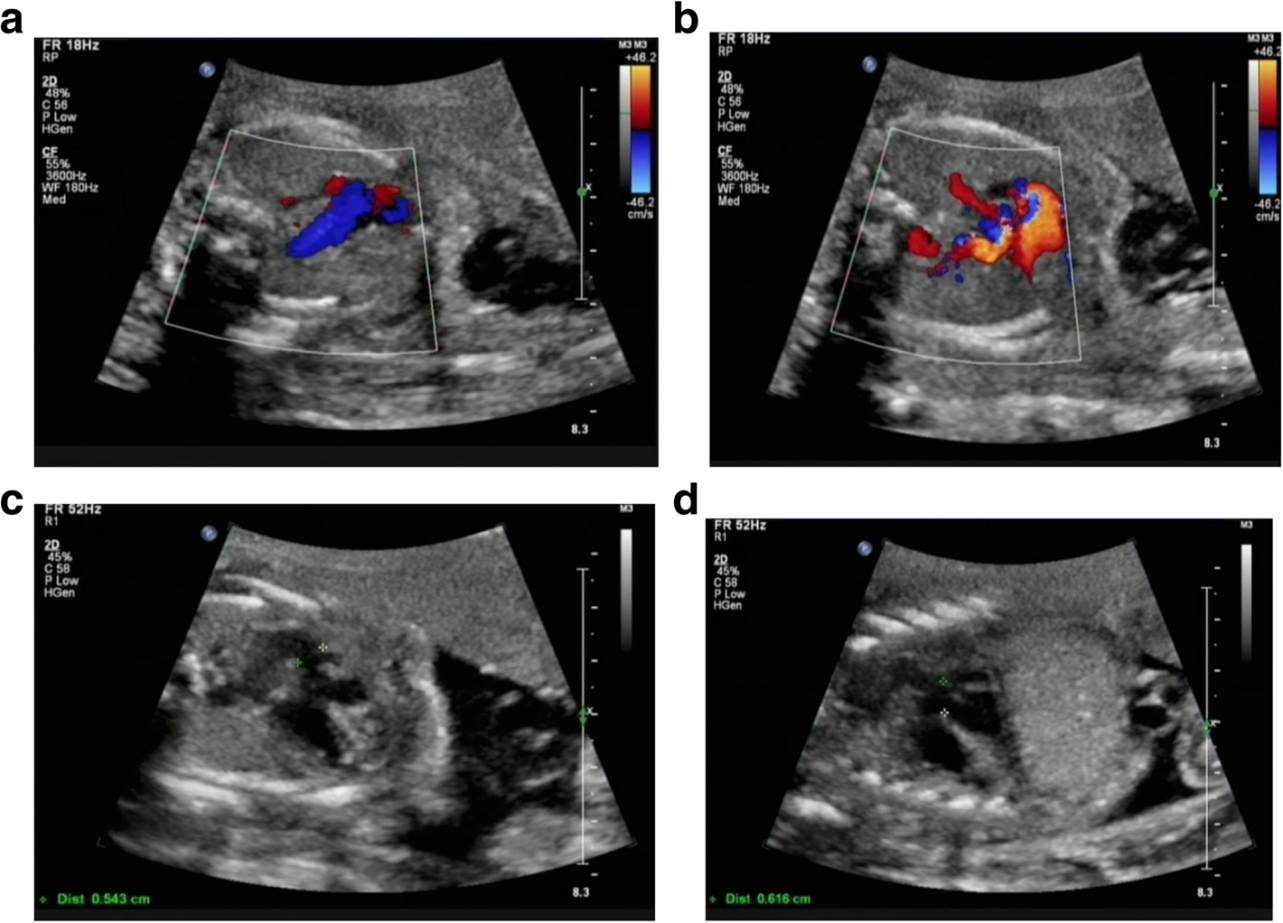
fetal echocardiography of the second fetus. **a** three vessels views in fetal echocardiography; **b** persistent trunk; **c** and (**d**) ventricular septal defect

One year later, the woman came to our center again during her second pregnancy at 21 weeks of gestation due to fetal congenital heart disease (CHD). The family history was negative for CHD. Her second pregnancy was uneventful before 21 weeks with the NT 1.9 mm at 11 weeks. However, the radiologist suspected fetal CHD after the first fetal anomaly scan at 21 weeks. Fetal echocardiography was offered shortly after the anomaly scan and revealed fetal ventricular septal defect, overriding aorta and persistent trunk (seen in Fig. 4a-d). The ultrasound findings of fetal anomaly scan also revealed increased nuchal fold (NF 9.3 mm) and fetal ascites. Once again, the fetal karyotyping and CNV-seq were done after signing the informed consent. No gross abnormalities identified in fetal karyotype (Fig. 1b). However, the abnormal CNV-seq results for the second fetus were: arr [GRCh37] 4p15.2p16.3 (40001–23,320,000) × 3, 4q32.3q35.2 (167040001–190,940,000) × 1, (Fig. 2b). The fetal karyotyping was reassessed as46, XY, der (4), (pter→q32.3p15.2 → pter), according to the ISCN (2016).Parental peripheral blood samples were tested by CNV-seq but no abnormalities were observed. Based on the two pregnancies, pericentric inversion of chromosome 4 in one of the parents was highly suspected. Fluorescence in situ hybridization (FISH) was suggested, but was declined. Parents decided to terminate the pregnancy at 24 weeks of gestation.

#### Chromosome analysis

Amniocentesis was performed to obtain the fetal samples after signing the informed consents. Peripheral blood samples were collected from both parents. Chromosome analysis was performed according to the standard protocol using G-banding.

#### Copy number variation sequencing (CNV-seq)

DNA libraries were constructed by transposase to fragment and add tag to each end of DNA fragments, and PCR amplified molecules subjected to massively parallel sequencing on the NextSeq 500 platform (Illumina, US). Plots of log2 [mean CN ratio] per bin (Y-axis) versus each 20 kb bin (X-axis) were generated for each of the 24 chromosomes. For reference, a log_2_ of 0 indicates a CN of 2.0 (disomy) while log_2_ values of 1.5 and 0.5 indicate a CN of 3.0 (duplication) and a CN of 1.0 (deletion), respectively. For reporting CNVs, CN ranges of 2.9–3.1 for a duplication and 0.9–1.1 for a deletion were used.

## Discussion

Recombinant chromosome 4 syndrome (rec 4 syndrome) is a very rare genetic condition, primarily caused by a pericentric inversion of chromosome 4 in a parent [2, 7]. A total of 18 rec (4) syndrome cases have been well-documented, showing different recombinant types and varying clinical presentations (17 in literature and 1 in DECIPHER database, Table 1) [5, 7–20]. Generally, a pericentric inversion will give rise to four types of gametes during the meiosis, including two balanced and two unbalanced [18, 21]. Balanced gametes, either the normal chromosome or the same inverted chromosome inherited from the parent, will generally develop into normal fetuses. However, unbalanced gametes are rather complex issues. During meiosis in carriers, a chromosome containing a large inverted segment and its normal homolog are predicted to form a homosynaptic inversion loop, in order to obtain optimal pairing of the matching segment. Any odd number of crossovers within the inversion loop leads to the production of two alternate recombinant chromosomes: in one chromosome the distal part of the short arm is duplicated and the distal part of the long arm is deleted; the opposite occurs to be short arm deletion and long arm duplication [7]. In our study, although FISH was refused by the parents, the constitutional chromosomal abnormalities occurred in two fetuses are most probably consistent with recombinant (4) syndrome. The breakpoints on long arm of chromosome 4 for two fetuses in the present study were different from any other previously reported cases. To the best of our knowledge, although it is within the range of 4q28-4q35, it’s the first time that the breakpoint of 4q32.3 among rec (4) syndrome cases is going to be reported.

**Table 1:** clinical presentations of two types of recombinants

Authors	Sub-bands	Clinicalpresentations					Outcome and annotations
		Facial dysmorphisism	Growth and development/mental delay	CHD	Extremities abnormailities	Genital abnormalities	
Dup 4q and del 4p	(10 cases)						
Narahara et al. 1984 [9]	p15.2q35	Microcephaly frotal bossing hypertelorism epicanthic folds small chin	Growth retardation development delay	VSD	Sacral dimple	No	Live
de la Flor & Guitart 1987 [10]	p16q31.3	Broad flat nasal bridge high forehead hypertelorism small chin (Greek Helmet appearence)	Growth retardation development delay	No	No	No	Live
Hirsch et al.1993 [11], patient III-1	p15.32q35	Flat nasal bridge prominent forhead small chin hypertelorism microcephaly iris coloboma retinal dysplasia	Growth retardation development delay	No	No	No	Live
Wolf et al. 1994 [12]	p13q28	NA	NA	NA	NA	NA	Fetal demise
Villa et al.1995 [13]	p15.2q28.2	Greek warrior helmet appearance prominent forhead hypertelorism downslanting palpebral fissures epicanthal folds small chin	Growth retardation	PDA	Abnormal fingers and clubfeet	Cryptorchidism	Redundant skin on the neck, arm and back neonatal death
Ogle et al.1996 [14]	p15.2q35	Consistent to the WHS high forhead broad nasal bridge downslanting palpebral fissures abnormal ears	Growth retardation development delay intelectual disability	Small VSD	Thoracic scoliosis joint contractures abnormal fingers	Secondary sexual characteristics were underdeveloped, the left testis was in the scrotum and hypoplastic, and the right undescended.	Live
Mun et al. 2010 [15]	p16q31.3	No	Mild growth retardation	No	No	No	Live
Dufke et al. 2000 [16]	p16.2q35.1	Consistent to WHS high forehead hypertelorism broad nasal bridge dolichocephaly	Growth retardation	No	No	No	Live
Malvestiti et al. 2013 [17]	p16.3q35.2	Hypertelorism prominent eyes low-set ears beaked nose small chin	Intrauterine growth retardation	No	No	No	Terminated at 20 weeks of gestation
Our fetus 1	p15.2q32.3	NA	Intro uterine growth retardation	No	No	No	Increased NT, ascites, terminated at 24 weeks of gestation
Dup 4p and del 4q	10 cases						
Hirsch et al.1993, [11] patient II-5	p15.32q35	Unilateral ptosis facial asymetry prominent ears with abnormal helices	Mental retardation but was reported to be caused by birth asphyxia	No	Congenital hip dis-location and scoliosis	No	Live
Battaglia et al. 2002 [5]	p14q35.1	Mild ptosis, upturned nose, thin upper lip prominent ears with a mild cupped configuration	Growth delay	Congenital heart defectbut not mentioned in detail	Short fingers with transverse creases, abnormal toe, coccyx dimple	Underdeveloped scrotum	Live
Garcia-Heras et al. 2002 [18]	p15q35	Microcephaly prominent forhead shallow orbit midface dysplasia small chin	Growth and develoment delay	Pulmonary hypertension and PDA	No	No	Live
Stembalska et al. 2007 [19] patient1	p14q35	Microcephaly abnormal ears with cupped configuration broad nose short neck	Growth and development delay	No	Short fingers	No	Live
Stembalska et al. 2007 [19] patient 2	p14q35	Microcephaly abnormal ears with cupped configuration broad nose short neck	Growth and development delay	No	Short fingers	No	Live
Maurin et al. 2009 [20]	p15.1q35.1	Anteverted nose large philtrum downslanting palperbral fissure thin upper lip short neck	Growth and development delay	Interauricular septal defect	Mild edema of feet	No	Live
Hemmat et al. 2013 [7]	p15.1q35.1	Microcephly broad nose with anteverted nares thin upper lip abnormal ears short neck	Developmental delay	Congenital heart disease but not mentioned in detail	No	Yes but not mentioned in detail	Live
Tassano et al. 2012 [8]	p15.1q35.1(de novo)	Hypertelorism prominnet ears with cupped configuration saddle nose thin upper lip retrognathia short neck	Growth and development delay	No	Congenital lucation of the right hip bilateral clubfeet	No	Live
Decipher Patient ID 269158	p15.3q34.2	Prominent forhead	Developmental delay	ASD	NA	NA	Live
Our fetus 2	p15.2q32.3	NA	No	VSD overriding aorta persistent trunk	No	No	Terminated at 24 weeks of gestation

*No* no such clinical manifestation was present, *NA* not available, *CHD* congenital heart disease, *VSD* ventricular septal defect, *PDA* Patent ductus arterious, *ASD* Atria septal defect

Ten cases in upper part of Table 1 harbered the recombinant type of Dup 4q and del 4p. Ten cases in lower part of Table 1 harbered the the recombinant type of Dup 4p and del 4q

According to the previous conclusion, duplicated segments are always longer than deleted ones in viable inv. dup del cases [6, 12, 13]. It sounds reasonable because a large deletion might be more deleterious than a large duplication. Embryos with a large deletion are most likely to suffer spontaneous miscarriages in the very early gestational ages. However, through literature review, we found three viable rec (4) cases with the recombinant type of a large deletion and a small duplication [9, 11, 14]. This finding might be explained by the vision that individuals have different tolerance to genetic deletions. Interestingly, we found all three viable cases with large deletion carried a large 4p deletion and a small 4q duplication. However, there was no viable rec (4) case with a large 4q deletion and a small 4p duplication. This observation suggests that large 4q deletion might be more deleterious than 4p deletion. Large 4q deletion might be lethal in early embryonic development and then lead to spontaneous miscarriages.

Rec (4) syndrome is primarily caused by the pericentric inversion from one of the parents. But, it is worth noting that, not all rec (4) syndrome cases were derived from the parental pericentric inversion of chromosome 4. Tassano et al. [8] reported the first de novo rec (4) syndrome case with dup 4p at p15.1 and del 4q at q35.1 in 2012. Besides the possibility of the presence of a cryptic inversion undetectable by classical cytogenetic or FISH analysis on one chromosome 4 of the parents, the second possible mechanism might be that inverted low copy repeats in the same chromosome arm form a partial folding of one homologue onto itself with a recombination event between the inverted repeats. The pre-meiotic double-strand breaks with subsequent fusion between sister chromatids was also the third possible mechanism that they suggested [6, 8]. In addition to these hypothesizes, harboring chromosome 4 inversion gametes because of germline mosaicism should also been considered.

The clinical phenotype of rec (4) has been a subject of debate for years. Although previous studies have conducted the genotype-phenotype correlation of rec (4) syndrome, the results were contradictory, with some authors suggested that rec (4) syndrome appears to be an entity which can be suspected on the basis of specific clinical features [5, 7], while others argued that rec (4) syndrome is not characterized by a recognizable phenotype [18]. The previous studies were unable to point out the genotype-phenotype relationship because they described the rec (4) syndrome as an entirety. We recommend classifying it into two categories according to its two recombinant types: dup 4p with del 4q, and dup 4q with del 4p, in order to better describe the genotype-phenotype correlation.

The clinical manifestations of rec (4) cases with dup 4q and del 4p are consistent with WHS, i.e., 4p- syndrome. There were 10 cases with dup 4q and del 4p through literature review (shown in the upper part of Table 1). All cases presented with an apparent “Greek Warrior Helmet appearance”, which is a typical clinical feature of WHS, including prominent forehead, flat and broad nasal bridge, hypertelorism, small chin, epicanthic folds, etc. All cases presented with different degrees of growth retardation and development delay, even the prenatal case, our case 1 presented early onset intrauterine growth retardation. Growth retardation and development delay are also the most common clinical features of WHS. The incidence of congenital heart disease in patients with recombinant type of dup 4q and del 4p in Table 1 was approximately 30%, which is similar to WHS patients [22, 23].We proposed that the main clinical features of rec (4) cases with dup 4q and del 4p were caused by 4p deletion, rather than 4q duplication. The expression effect of the 4p deletion might be stronger than that of some simultaneous partial duplication. Some authors observed that patients with a very small 4p16.3 deletion combined with a large concomitant duplication of proximal segment of short arm of chromosome 4 presented with typical WHS clinical features [24, 25]. In Table 1, the two patients in Dufke and Malvestiti et al.’s studies carried a large 4q duplication with a very small 4p deletion. Both patients presented with typical WHS clinical manifestations [16, 17]. Our findings provided more evidence to support the opinion of “stronger gene effect of 4p deletion” .

The craniofacial dysmorphisms in cases with dup 4p and del 4q are not very consistent (10 cases shown in the lower part of Table 1). However, the prominent clinical manifestation among these patients is the high incidence of congenital heart disease (CHD). Six of 10 cases presented with CHD, giving a very high incidence of 60% (including the Decipher patient 269,158 and our fetus 2) [5, 7, 18, 20]. It has been reported that pure 4p terminal duplications with the same size on short arm of chromosome 4 seldom give rise to CHD [2]. However, cases with pure 4q terminal deletions or rec (4) cases with dup 4p and del 4q have much higher incidence of CHD (approximately 50%) [2, 7]. Our data provide supporting evidence that 4q terminal might be a candidate region involved in heart development. The smallest overlapping region (SOR) of the six CHD cases is the 4 Mb region proximal to the telomere on the long arm of chromosome 4 (sub-band of 4q35.2). There are five Ref Genes encompassed in the SOR, including *FAT1*, *ZFP42*, *TRIML2*, *FGR1* and *DBET*. We suggest *FAT1* might be the putative gene associated with cardiovascular development. According to the Project of Tissue-specific circular RNA induction of human fetal development, this gene presents much higher level of expression in fetal heart as early as 10 weeks of gestation, compared with other organs. This gene also expresses high levels in aorta and coronary arteries. Although there are no reports concerning correlation between *FAT1* and cardiovascular disease, some authors found *Fat1* may control vascular smooth muscle cell functions by facilitating migration and limiting proliferation [26]. It has been reported that *FAT1*intracellular domain can interact with multiple mitochondrial proteins and regulate cell growth and metabolism [27]. The facts of its high expression levels in fetal heart as well as its ability to regulate mitochondrial function led us propose *FAT1* to be a candidate gene involved in cardiovascular development.

## Conclusions

The present study showed a novel breakpoint on the long arm of chromosome 4 among the rec (4) syndrome cases. Genotype-phenotype correlation analysis reveals that the clinical manifestations of rec (4) cases with dup 4q and del 4p resemble those of WHS. High incidence of congenital heart disease is an obvious feature among dup 4p and del 4q cases, suggesting the putative association of 4q35.2 deletion with cardiovascular disease.
